# Forsythia Fruit Prevents Fulminant Hepatitis in Mice and Ameliorates Inflammation in Murine Macrophages

**DOI:** 10.3390/nu13082901

**Published:** 2021-08-23

**Authors:** Yun Hee Jeong, Youn-Hwan Hwang, Tae In Kim, You-Chang Oh, Jin Yeul Ma

**Affiliations:** Korean Medicine (KM)-Application Center, Korea Institute of Oriental Medicine, 70, Cheomdanro, Dong-gu, Daegu 41062, Korea; runxi0333@kiom.re.kr (Y.H.J.); hyhhwang@kiom.re.kr (Y.-H.H.); tikim@kiom.re.kr (T.I.K.)

**Keywords:** Forsythia Fruit, liver injury, inflammation, antioxidant, lipopolysaccharide, D-galactosamine

## Abstract

Forsythia Fruit (FF), the fruit of *Forsythia suspensa*, has been used since ancient times as an herbal medication in East Asia to treat inflammation, gonorrhea, and pharyngitis. However, the efficacy of FF against liver damage due to inflammation has not been studied. Here, we explored the protective effects of FF in a mouse hepatitis model induced by lipopolysaccharide (LPS)/D-galactosamine (GalN) treatment. We measured inflammatory cytokine and aminotransferase levels in mouse blood and analyzed the effects of FF on inflammatory gene and protein expression levels in liver tissue. Our results show that FF treatment effectively lowers inflammatory cytokine and serum aminotransferase levels in mice and inhibits the expression of hepatic cytokine mRNA and inflammatory proteins. Furthermore, treatment with FF activated the antioxidant pathway HO-1/Nrf-2 and suppressed severe histological alteration in the livers of LPS/D-GalN-treated mice. Further investigation of the effects of FF on inflammatory reactions in LPS-stimulated macrophages showed that pretreatment with FF inhibits inflammatory mediator secretion and activation of inflammatory mechanisms both in a mouse macrophage RAW 264.7 cells and in primary peritoneal macrophages. These results show that FF has potential worth as a candidate for the treatment of fulminant inflammatory reactions and subsequent liver injury.

## 1. Introduction

Fulminant liver injury is characterized by rapid, widespread liver dysfunction and can result in encephalopathy, jaundice, and severe coagulopathy [1,2]. It is also a clinical manifestation of sudden and severe hepatic failure, which is difficult to prevent and treat, resulting in poor prognosis and a high mortality rate [3]. The main causes of acute liver injury are antigen-induced infections and poisoning by hepatotoxic drugs, but there are also many unknown causes [2]. At present, the only effective treatment is liver transplantation, so the development of effective prevention and treatment modalities are necessary [4].

Lipopolysaccharide (LPS) is an endotoxin originated from the gram-negative bacteria *E. coli* and was initially confirmed as a Toll-like receptor 4 (TLR4) ligand, which causes a rapid and powerful inflammatory reaction leading to sepsis or multiple organ failure [5]. In addition, LPS plays a pivotal role at the onset of endotoxic damage and increases inflammatory cytokine expression, causing liver damage. D-galactosamine (GalN) decreases the concentrations of uridine triphosphate, uridine diphosphate, and uridine monophosphate through metabolic disorders of galactose, leading to the inhibition of RNA synthesis, infiltration of inflammatory cells, necrosis of liver cells, and induction of lesions similar to hepatitis [6,7]. D-GalN also induces changes in colorectal mucosal permeability, increasing endotoxin absorption, which interferes with the ability of liver cells to repair membranes and causes hepatic toxicity [8]. Eventually, D-GalN causes necrosis of the liver during acute exposure and cirrhosis of the liver and cellular tumors during chronic exposure [9,10].

Therefore, D-GalN increases the reactivity of the liver toward endotoxins including LPS, resulting in acute hepatic toxicity within hours, and models of acute hepatic damage caused by LPS/D-GalN show hepatocyte necrosis and apoptosis. Thus, LPS/D-GalN is widely used in studies related to the mechanisms underlying hepatic damage and drug development [11]. Reactive oxygen species increased by LPS/D-GalN activate macrophages in liver tissue, and the activated macrophages produce inflammatory mediators including tumor necrosis factor (TNF)-α, interleukin (IL)-6, and IL-1β cytokine [12]. These inflammatory cytokines induce hepatocyte necrosis and reduce antioxidant enzyme activity [13]. Consequently, inhibition of inflammatory cytokines and activation of antioxidant enzymes are important factors for the treatment and prevention of acute liver damage caused by LPS/D-GalN.

Inflammation is the central defensive mechanism against external stimuli such as microbial or viral infection, injury, and exposure to endotoxin and is initiated through the activation of microglia and macrophages [14]. Macrophages play a dispensable role in controlling inflammation and produce inflammatory mediators in response to external causes such as LPS [15]. In macrophages with TLR4 activation, inflammatory mechanisms such as nuclear factor (NF)-κB, activator protein (AP)-1, and mitogen-activated protein kinase (MAPK) are also induced, and the expression of inflammatory synthetic enzyme inducible nitric oxide synthase (iNOS) and secretion of nitric oxide (NO) are increased [16,17]. However, the inflammatory reaction is also effectively inhibited by the activation of the antioxidant mechanism nuclear factor erythroid 2-related factor 2 (Nrf-2) and heme oxygenase (HO)-1. HO-1 inhibits the secretion of NO, TNF-α, IL-6, and IL-1β as an important regulator of the inflammation and is strongly induced by macrophages [18]. HO-1 expression directly inhibits the production of NO and iNOS and is controlled by the redox-sensitive transcription factor Nrf-2, which regulates various antioxidant enzymes [19]. When the inflammatory response is activated, Nrf-2 translocates to the nucleus and combines to the antioxidant response element to induce HO-1 [19]. Thus, many anti-inflammatory agents act via enhancing HO-1 production via Nrf-2 activation. In addition, mouse peritoneal macrophages are retained within the mouse abdominal cavity by thioglycollate medium and are often used to confirm the efficacy of in vitro inflammation studies [20].

FF is an herbal medicine that has been widely used for a long time in East Asia to treat inflammation, gonorrhea, and pharyngitis [21]. A previous study demonstrated that FF had anti-microbial effects on membrane permeability and apoptosis in *Salmonella* [22]. In addition, another study reported that FF showed anti-diabetic and anti-hyperlipidemic effects in a streptozotocin-induced diabetes mouse model [23]. Recently, in addition to the pharmacological efficacy of FF, studies on its applicability as a functional food considering nutritional properties have also been reported. FF is rich in vitamin P, and the effect of inhibiting lipid peroxidation in high-cholesterol diet rats through antioxidant action has been reported [24]. In addition, FF was studied for its applicability as a feed additive for effective fattening by reducing the risk of peroxidation in broiler chickens and increasing nutrient digestibility and growth performance in a stress situation due to high temperature [25]. However, the effects of FF on liver damage in mice and on the inflammatory reaction in macrophages and the regulation of FF on its associated mechanisms have not been studied before. Therefore, we investigated the protective efficacy of FF against LPS/D-GalN-induced fulminant hepatic failure and explored how FF impacts related molecular mechanisms. Furthermore, we tested the inhibitory efficacy of FF against the inflammatory reaction in an LPS-stimulated mouse macrophage RAW 264.7 and primary macrophages.

## 2. Materials and Methods

### 2.1. Plant Material

FF was obtained from Yeongcheonhyundai Herbal Market (Yeongcheon, Korea) and was identified by Prof. KiHwan Bae (Department of Pharmacy, Chungnam National University, Korea). Dried FF (50.0 g) was extracted by heating at 100 °C for 3 h using 1 L distilled water (DW) (Daewoong extractor, Daewoong, Seoul, Korea). Extract solution was filtered using 150 μm sieve, freeze-dried, and stored at −20°C until use. The yield was 12.58%.

### 2.2. Materials and Reagents

055:B5 LPS from *E. coli* and D-GalN were acquired from Sigma (St. Louis, MO, USA). Enzyme-linked immunosorbent assay (ELISA) antibody kits were obtained from Thermo (Rockford, IL, USA). Extraction kits for RNA isolation were acquired from iNtRON (Sungnam, Korea). Synthesizing kits for DNA and Master Mix for qPCR were acquired from Bioneer (Daejeon, Korea). Oligonucleotide primers were synthesized by Bioneer. Western blotting antibodies were obtained from Cell Signaling (Boston, MA, USA). Cell culture reagents, including antibiotics, fetal bovine serum (FBS), and Roswell Park Memorial Institute (RPMI) 1640 were obtained from HyClone (Logan, UT, USA). Dexamethasone (Dex) and bovine serum albumin (BSA) were purchased from Sigma. Cell-counting kits (CCK) were acquired from Dojindo (Kumamoto, Japan). Standard compounds, forsythoside A, pinoresinol, and phillygenin were purchased from Chem Faces (Wuhan, china). High-performance liquid chromatography (HPLC)-grade methanol was purchased from Merck (Darmstadt, Germany). ACS reagent-grade acetic acid was obtained Sigma. All water solutions were using a Puris-Evo RO water system (Mirae ST Co., Ltd., Anyang, Korea). HPLC analysis samples were filtered through 0.2 μm membrane filters before use.

### 2.3. Experimental Animals

Six weeks old male “imprinting control region” (ICR) mice (30 ± 3 g each) were acquired from Samtako BioKorea (Osan, Korea). All mice were acclimatized for 7 days and were maintained at a room temperature (RT) under a 12 h:12 h light/dark cycle with ad libitum. The mice were subjected to overnight fasting before injection of hepatitis inducers. All experimental procedures in this animal study were carried out depending on the guidelines of the Korea Institute of Oriental Medicine (KIOM)’s Animal Care and Use Committee (Reference number #D-17-020).

### 2.4. Fulminant Hepatitis Mice Model by LPS/D-GalN Injection

Briefly, the mice were sorted randomly into four groups (normal controls, LPS/D-GalN, FF 100 mg/kg + LPS/D-GalN, and FF 300 mg/kg + LPS/D-GalN; *n* = 9 each). Treated mice were orally administered FF once a day for 6 days and intraperitoneally injected with 50 μg/kg LPS and 1 g/kg D-GalN on the last day. Six hours after LPS/D-GalN injection, the animals were anesthetized with isoflurane gas and blood was collected via puncture of the abdominal vena cava. Blood serum was obtained by centrifuging the blood at 2000× *g* for 15 min. Livers were collected and gently rinsed with phosphate-buffered saline (PBS). Serum cytokine levels were measured with ELISA antibodies. The serum levels of alanine aminotransferase (ALT), aspartate aminotransferase (AST), and alkaline phosphatase (ALP) were determined by an XL-200 automatic clinical chemistry analyzer (Erba, Mannheim, Germany).

### 2.5. RNA Extraction, DNA Synthesis, and Real-Time Reverse Transcription-Polymerase Chain Reaction

Isolated total RNA (1 μg) from liver tissue were used for synthesis of cDNA. Sequences of oligonucleotide primer are indicated in Table 1, and real-time reverse transcription-polymerase chain reaction (RT-qPCR) was conducted in accordance with a previously described method [20]. Forty PCR cycles were run using the QuantStudio 6 Flex Real-time PCR System (Thermo), and the samples were compared through the relative CT method.

### 2.6. Histopathological Analysis

Tissue samples from mouse livers were rinsed with PBS and were fixed in a 10% formaldehyde solution. Liver tissues were then dehydrated in 70–100% ethanol aqueous solution and embedded in paraffin. Paraffin blocks were cut to a thickness of 5 µm by rotary microtome (RM 2165, Leica, Wetzlar, Germany) and were stained using hematoxylin and eosin (H&E). Liver injury in these sections was observed with an Axioskop 40 (Oberkochen, Germany) and was taken at 400× magnification.

### 2.7. Preparation of Protein Extracts and Western Blot Analysis

The liver tissue samples and macrophage cells were lysed in radioimmunoprecipitation assay buffer (Millipore, Bedford, MA, USA) for total cell protein or in NE-PER extraction reagent (Thermo) for cytosolic and nuclear proteins. Concentrations of total protein were measured by Bradford protein assay reagents (Bio-Rad, Hercules, CA, USA). Equal amount of proteins was separated and then blotted in accordance with a previously described method [20]. Proteins on the membrane were blocked and then incubated with various primary antibodies followed by secondary antibodies (Table 2). Immunoreactive bands of target protein were detected using enhanced chemiluminescence solution (Bio-Rad). Each detected protein band was normalized by internal control proteins and was quantified using ImageJ software (version 1.53k).

### 2.8. Culture of Macophage Cell Line

RAW 264.7 macrophages were acquired from American Type Culture Collection (Manassas, VA, USA) and were cultured using RPMI 1640 medium containing 10% FBS and 1% antibiotics in a CO_2_ incubator. The cells were stimulated by incubating in fresh RPMI 1640 media containing 200 ng/mL LPS in the presence or absence of pretreated FF.

### 2.9. Isolation and Culture of Mouse Peritoneal Macrophages

Following intraperitoneal injections of 3% sodium thioglycollate medium (1 mL), five male ICR mice were housed per cage in a 12 h:12 h light/dark cycle. Four days after the injections, the mice were sacrificed and peritoneal macrophage cells (PMC) were collected by flushing with PBS. Red blood cell lysis buffer was then added to the cell suspensions in PBS, after which the samples were incubated for 5 min at RT. After centrifugation at 500× *g*, the supernatants were discarded and PMC were suspended in fresh RPMI 1640 medium and incubated with or without FF under the same conditions as those used for RAW 264.7 cells. All experimental procedures for isolation of mouse PMC were carried out depending on the guidelines of the KIOM’s Animal Care and Use Committee (Reference number #D-17-001-1).

### 2.10. Cell Viability Assays

Macrophage viability was examined using CCK reagent in accordance with a previously described method [20]. Briefly, macrophages were pre-treated with FF for 24 h, and CCK solution was added, after which the samples were incubated for additional 1 h. The absorbance was then measured at a wavelength of 450 nm using microplate reader (SpectraMax i3, Molecular Devices, San Jose, CA, USA).

### 2.11. Measurement of NO and Inflammatory Cytokine Secretion

NO and inflammatory cytokines were measured under the same conditions as in the previous study [20]. Cultured macrophages were pre-treated with FF, stimulated with LPS after 1 h, and incubated for an additional 24 h. NO was detected with Griess reagent and absorbance was measured at 570 nm, and the secretion of inflammatory cytokines in the culture media was quantified by ELISA.

### 2.12. HPLC Instrument

HPLC system was set up column oven, an auto sampler, a binary pimp and UV/VIS detector (Dionex Ultimate 3000 system, Dionex Corp., Sunnyvale, CA, USA). All analysis data was processing using Chromeleon 7 software (Thermo, Waltham, MA, USA).

### 2.13. Preperation of Standard and Sample Solutions

The FF was dissolved in water at 5 mg/mL concentration using ultrasonicator (JAC Ultrasonic JAC-3010, Hwaseong, Korea) and after extraction, extract was filtered with a 0.2 μm membrane. 10 μL of extract solution was injected for HPLC analysis. Standard solutions of forsythoside A, pinoresinol, and phillygenin was prepared at 1.0 mg/mL (1000 ppm) using methanol and stored at 4 °C until use. For HPLC analysis, each compound standard solution was diluted with methanol at each standard curve concentration.

### 2.14. HPLC Analysis Method

HPLC analysis was conducted to identify of contents of three compounds (forsythoside A, pinoresinol, and phillygenin) in FF. HPLC analysis was performed using X bridge C18 column (250 mm × 4.6 mm, 5 μm) connected to a C18 guard cartridge (4.0 mm × 3.0 mm). The mobile phase was eluted at Flow rate 1 mL/min with gradient of 0.3% acetic acid in water (eluent A) and methanol (eluent B). Gradient eluted method was applied: 0–8 min, 5–30% B; 8–24 min, 30–57% B; 24–39 min, 57–60% B; 39–50 min, 60–70% B; 50–60 min, 70–100% B. The HPLC condition was follows: chromatogram data was detected at 280 nm, the injection volume was 10 μL and temperature of column and auto sampler was keep 40°C and 20°C, respectively (Table 3). Calibration curves, assessed by standard solution and the limits of detection (LOD) and quantification (LOQ) under the chromatographic conditions, were determined by injecting a series of standard solutions. Each samples were three injected under same condition and data was processed using Chromeleon 7 software (Thermo).

### 2.15. Statistical Analysis

All experimental results are expressed as means ± standard error of the mean. Statistical significance was determined by one-way analysis of variance followed by Dunnett’s test after comparing each treatment group. Statistical significance was defined as *p* < 0.05.

## 3. Results

### 3.1. Content of Major Compounds of FF

**Figure 1:**
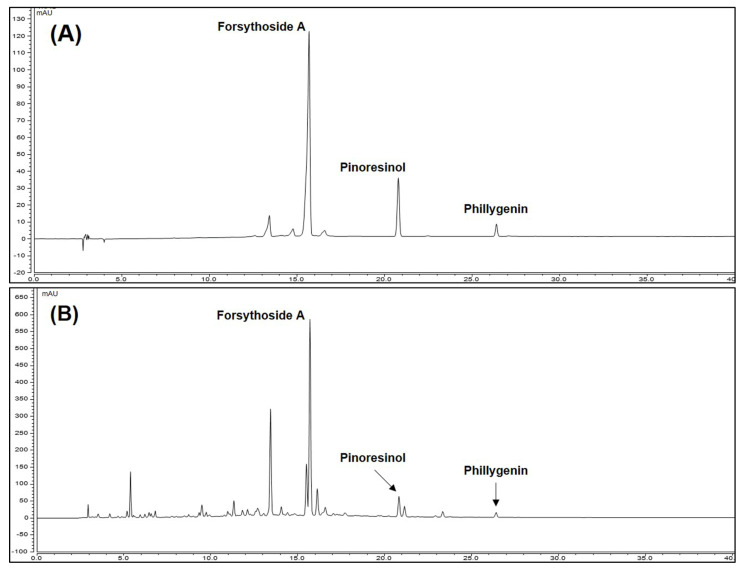
High-performance liquid chromatography chromatograms of standard solution (**A**) and FF (**B**) at 280 nm.

We conduct HPLC analysis to confirm that contents of three compounds forsythoside A, pinoresinol, and phillygenin in FF that show bioactivity. Each component was selectively detected and identified under HPLC-UV analysis method we established, consistent with a previous study [26]. The calibration curves the three compounds (forsythoside A, pinoresinol, and phillygenin) were y = 0.2516x − 3.8826, y = 0.1132x + 0.1922 and y = 0.1927x + 0.0909 with coefficients of determination of 0.9958, 0.9990, and 0.9994 at injected concentration ranges (Table 4). These result showed that calibration curve of three marker compounds has good linearity at the tested concentration range. To confirm the three compound were showed in FF, we compared the retention time and the UV spectrum of FF extract and each standard solution (Figure S1). As a result, the three compounds exhibited the same retention time 15.70, 20.82, and 26.40 min in FF (Figure 1). The area mean value of FF was calculated for each compounds calibration curve equation. The content of forsythoside A, pinoresinol, and phillygenin and were 4.54, 1.17, and 0.84% respectively. Forsythoside A was most abundant constituent in FF and we suggest that it was marker compound in FF.

### 3.2. Regulatory Effects of FF on Serum Cytokine and Aminotransferase Levels in LPS/D-GalN-Induced Hepatitis in Mice

**Figure 2:**
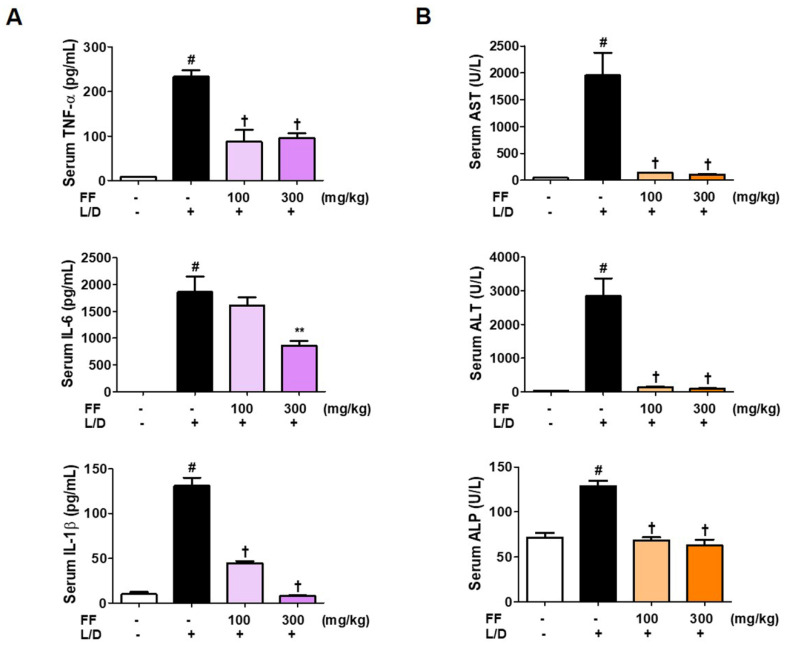
Effects of Forsythia Fruit (FF) on serum cytokine and aminotransferase levels in a lipopolysaccharide (LPS)/D-galactosamine (GalN)-induced hepatitis mouse model. Mice were pretreated with FF (100 and 300 mg/kg) or vehicle once per day for 6 days and 1 h before an LPS/D-GalN injection. After 6 h, blood was collected by abdominal vena cava puncture and serum was prepared by centrifugation. (**A**) Serum cytokine levels were determined using enzyme-linked immunosorbent assay antibodies. (**B**) Serum aminotransferase was analyzed using an automated clinical chemistry analyzer. Data are expressed as mean ± standard error of the mean (*n* = 9). TNF, tumor necrosis factor; IL, interleukin; L/D, LPS/D-GalN; ALT, alanine aminotransferase; AST, aspartate aminotransferase; ALP, alkaline phosphatase. Statistical significance was defined as # *p* < 0.05 (vs. normal controls), ** *p* < 0.01, and † *p* < 0.001 (vs. LPS/D-GalN treatment).

Inflammatory cytokine levels are important measures of the severeness of inflammation. In addition, ALT, AST, and ALP are markers of hepatic damage. Therefore, we analyzed these parameters to investigate the extent of fulminant liver injury and the regulatory effects of FF. Serum cytokine, ALT, AST, and ALP levels were significantly elevated 6 h after LPS/D-GalN treatment. However, as shown in Figure 2A,B, in the groups administered with two doses of FF, inflammatory cytokine, ALT, AST, and ALP concentrations in the mice serum were sharply reduced. IL-6 and IL-1β levels in the serum decreased in a dose-dependently, and the other factors were strongly suppressed at both doses. The normal control group did not show any abnormal changes in these measures.

### 3.3. FF Protects Mice from Liver Injury and Regulates the Expression of Hepatic Cytokine mRNAs upon LPS/D-GalN Stimulation

**Figure 3:**
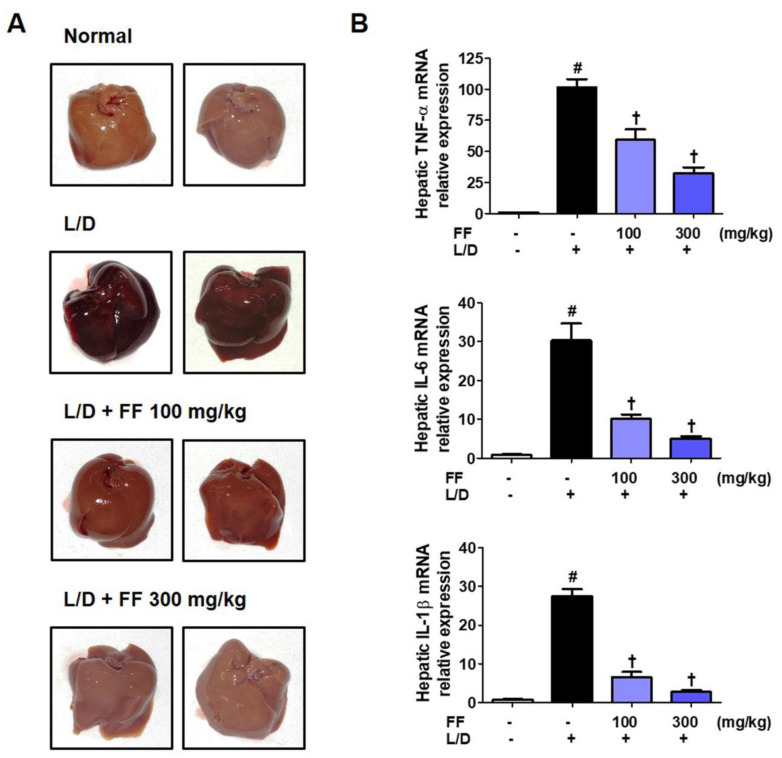
Effects of Forsythia Fruit (FF) on liver injury and expression of hepatic cytokines in lipopolysaccharide (LPS)/D-galactosamine (GalN)-induced hepatitis. Mice were pretreated with FF (100 and 300 mg/kg) or vehicle once per day for 6 days and 1 h before an LPS/D-GalN injection. After 6 h, mice were sacrificed, and livers were collected. (**A**) Images of hepatitis lesions in the mice. (**B**) mRNA levels of hepatic cytokines were analyzed by real-time reverse transcription-polymerase chain reaction. Data are expressed as mean ± standard error of the mean. L/D, LPS/D-GalN; TNF, tumor necrosis factor; IL, interleukin. Statistical significance was defined as # *p* < 0.05 (vs. normal controls) and † *p* < 0.001 (vs. LPS/D-GalN treatment).

Six hours after LPS/D-GalN was administered, the mice were killed and livers were collected. To determine the severity of liver injury of each group, liver images were taken. Livers in the LPS/D-GalN group mice suffered severe damage; in contrast, livers in the FF-administered group appeared to have a significantly improved pathology in a dose-dependent manner (Figure 3A). Furthermore, we extracted total RNA from these liver samples and analyzed the expression of inflammatory cytokines to determine how they are regulated by FF administration in liver tissue. Results showed that all cytokine mRNA within the liver tissue were strongly increased by LPS/D-GalN treatment, and they were dose-dependently significantly inhibited by FF administration (Figure 3B).

### 3.4. Hepatoprotective Effects of FF on Histopathological Changes and Regulatory Effects on the Inflammatioy Proteins Expression

**Figure 4:**
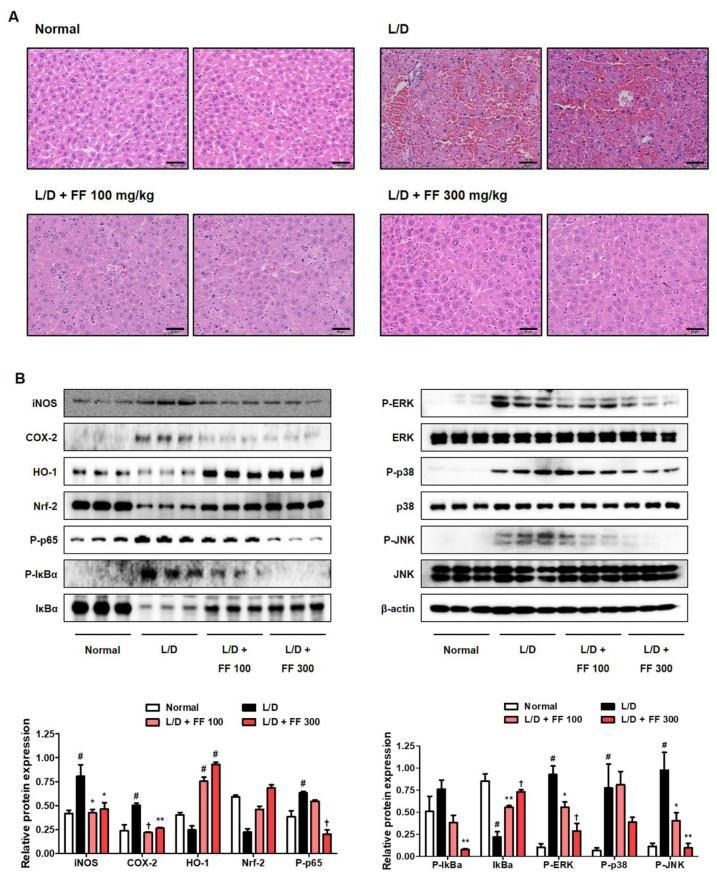
Effects of Forsythia Fruit (FF) on histopathological changes in the liver and activation of intracellular signaling molecules in lipopolysaccharide (LPS)/D-galactosamine (GalN)-induced hepatitis. Mice were pretreated with FF (100 and 300 mg/kg) or vehicle once per day for 6 days and 1 h before an LPS/D-GalN injection. After 6 h, mice were sacrificed, and livers were collected. (**A**) Hematoxylin and eosin staining of mouse liver. Scale bars = 50 μm. (**B**) Expression of inflammatory synthetic enzymes, inflammatory pathways, and antioxidant molecules were determined by Western blot analysis. The histograms show protein expression levels relative to those of a housekeeping protein. Data are expressed as mean ± standard error of the mean. L/D, LPS/D-GalN. Statistical significance was defined as # *p* < 0.05 (vs. normal control), * *p* < 0.05, ** *p* < 0.01, and † *p* < 0.001 (vs. LPS/D-GalN treatment).

The histopathological findings showed that LPS/D-GalN injection induced atrophy, hepatocyte necrosis, and infiltration of inflammatory cells. The fulminant changes observed in the LPS/D-GalN-injected mice significantly improved in those treated with 100 mg/kg FF, and the 300 mg/kg FF-administered group had no differences from the normal group (Figure 4A). Next, we analyzed the expression of inflammation-related proteins in the liver tissue. Cyclooxygenase (COX)-2 and iNOS, which are synthase proteins of prostaglandin (PG)E_2_ and NO, respectively, up-regulated in the LPS/D-GalN-administered group and significantly decreased in the FF-treated group, while the antioxidant mechanism protein HO-1/Nrf-2 showed opposite patterns (Figure 4B). P-NF-κB p65 and P-inhibitor of NF-κB alpha proteins were also strongly expressed in the liver tissue of the LPS/D-GalN-treated mice and were effectively suppressed in the FF-treated group (Figure 4B). Similarly, we observed that the phosphorylation of extracellular signal-regulated kinase, p38, and c-Jun NH_2_-terminal kinase proteins were effectively inhibited in the FF-treated group (Figure 4B).

### 3.5. Regulatory Effects of FF on the Secretion of Inflammatory Mediators and Activation of Inflammatory/Antioxidant Pathways in LPS-Stimulated RAW 264.7 Macrophages

**Figure 5:**
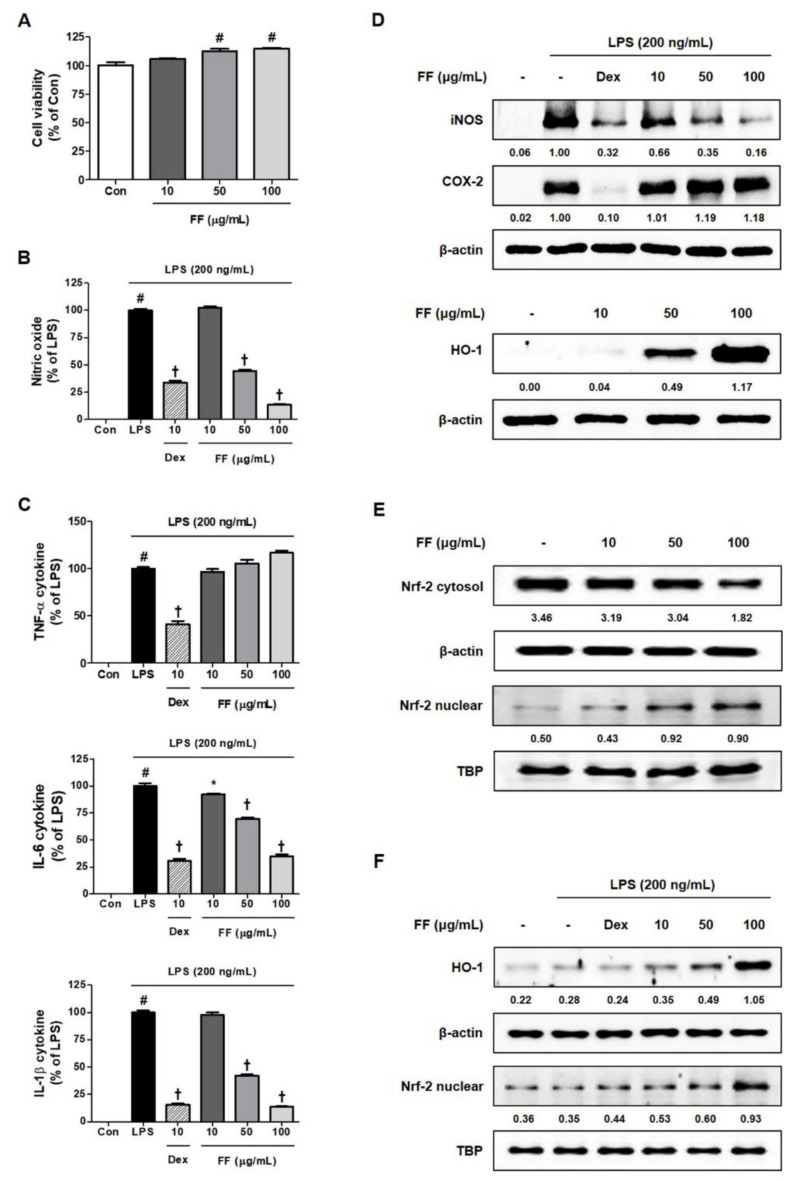
Effects of Forsythia Fruit (FF) on the secretion of inflammatory mediators and expression of intracellular pathway proteins in RAW 264.7 macrophages. Influence of FF on (**A**) cell viability, (**B**) nitric oxide secretion, and (**C**) cytokine production. (**D**) Expression of inducible nitric oxide synthase (iNOS), cyclooxygenase (COX)-2, and heme oxygenase (HO)-1, (**E**) nuclear translocation of nuclear factor erythroid 2-related factor 2 (Nrf-2), and (**F**) HO-1 activation and nuclear translocation of Nrf-2 under lipopolysaccharide (LPS) stimulation. Control cells were incubated with the vehicle alone. Data represent the mean ± standard error of the mean of the results from three independent experiments. Con, control; Dex, dexamethasone; TNF, tumor necrosis factor; IL, interleukin. # *p* < 0.05 (vs. controls), * *p* < 0.05, and † *p* < 0.001 (vs. LPS treatment).

Since the pathology of the acute hepatitis mouse model induced by LPS/D-GalN closely mirrored a fulminant inflammatory response, we investigated the influence of FF on the LPS-induced mouse macrophage-mediated inflammatory reaction. First, FF had little effect on RAW 264.7 macrophage viability (Figure 5A), effectively inhibiting the secretion of inflammatory mediators including LPS-induced NO and cytokines (Figure 5B,C). FF pretreatment also suppressed the expression of iNOS by LPS in macrophage cells, while high concentrations of FF treatment (over 50 μg/mL) induced antioxidant protein HO-1 expression (Figure 5D). Treatment with FF induced translocation into the nucleus from the cytoplasm of Nrf-2, which affected the activation of the antioxidant mechanism (Figure 5E). In addition, an investigation of the effects of FF on the activation of HO-1/Nrf-2 under LPS treatment showed that HO-1/Nrf-2 were activated at high concentrations of pretreatment with FF (Figure 5F).

### 3.6. Inhibitory Effects of FF on LPS-Induced Inflammatory Mediator Levels in Primary Macrophages

**Figure 6:**
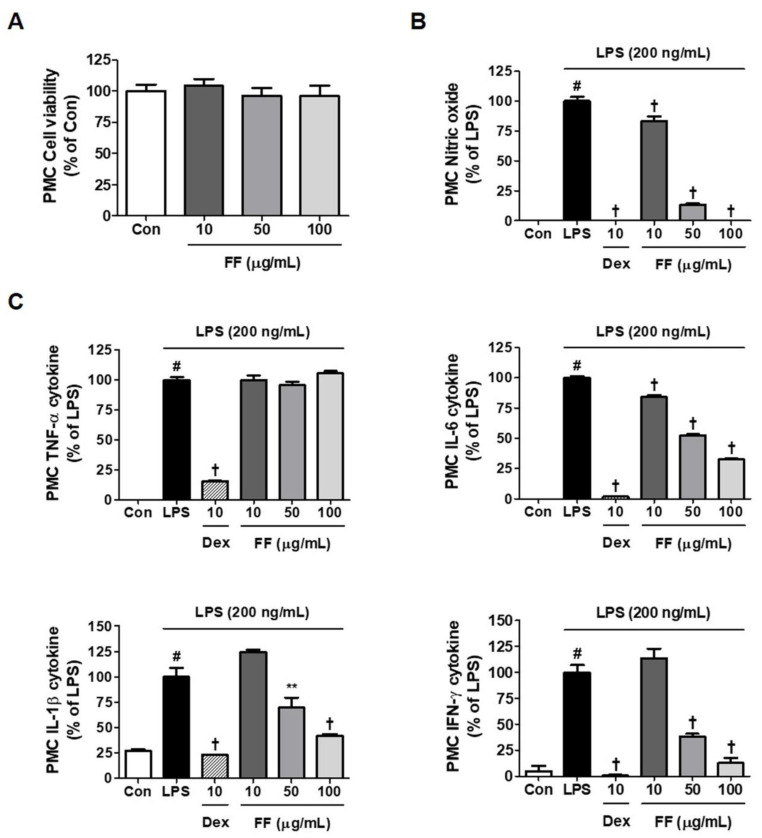
Effects of Forsythia Fruit (FF) on the production of inflammatory mediators in mouse peritoneal macrophage cells (PMC). Influence of FF on (**A**) cell viability, (**B**) nitric oxide secretion, and (**C**) inflammatory cytokine production. Primary macrophages obtained from five ICR mice were seeded on a 96- or 24-well culture plate and preincubated for 18 h. Then, the cells were pretreated with FF for 1 h and stimulated with lipopolysaccharide (LPS) for another 24 h. Control cells were incubated with the vehicle alone. Data represent the mean ± standard error of the mean of determinations from three independent experiments. Con, control; Dex, dexamethasone; TNF, tumor necrosis factor; IL, interleukin; IFN, interferon. # *p* < 0.05 (vs. controls), ** *p* < 0.01, and † *p* < 0.001 (vs. LPS treatment).

To confirm the inhibitory activity of FF on inflammatory response, we explored its effects on LPS-induced secretion of NO and inflammatory cytokine in primary macrophages. Treatment with FF did not exhibit cytotoxicity up to 100 μg/mL (Figure 6A), and it down-regulated the levels of NO and IL-6, IL-1β, and interferon-γ cytokines in a concentration-dependently (Figure 6B,C).

## 4. Discussion

Several previous studies have shown that fulminant hepatic injury is characterized by rapid and widespread liver dysfunction and is caused by antigen-induced infections, exposure to endotoxins, and poisoning [2]. Simultaneous injection of LPS and D-GalN dramatically increases the activity of endotoxin and its influence on liver tissue, so we used a mouse model to examine the efficacy of FF on protection of the liver in this study. LPS plays a critical role in the early stage of endotoxic damage, increases inflammatory cytokine levels, and damages liver tissue, so we explored the inhibitory activities of FF on inflammatory cytokine levels in mouse serum. High concentrations of aminotransferase are present in the liver and heart, and when liver parenchymal cells are damaged, ALT, AST, and ALP in the cytoplasm are released into the blood [27], so activity of these enzymes is an important indicator of liver injury. Thus, the concentrations of ALT, AST, and ALP in the mouse serum of each group were measured. We found that LPS/D-GalN treatment strongly induced the inflammatory cytokine and aminotransferase levels in the serum, and these were then efficiently reduced by FF administration (Figure 2).

Liver damaged by endotoxin or toxic substances has more surface bleeding due to severe hemorrhage [28], so morphological observation is used to determine the extent of liver damage. As shown in Figure 3A, we found that the severe hemorrhage caused by LPS/D-GalN injection significantly improved after FF administration. In addition, we confirmed that the expression of hepatic cytokine mRNA in liver tissue was also significantly and dose-dependently inhibited by FF treatment (Figure 3B). The histopathological changes examined with H&E staining also showed that FF treatment dramatically improved hepatic hemorrhage, hepatocytes necrosis, and inflammatory cell infiltration by LPS/D-GalN (Figure 4A).

In the model used in this study, the direct cause of liver damage was a fulminant inflammatory reaction by endotoxin, so we investigated inflammatory protein expression in liver tissue using immunoblotting. LPS activates MAPK/NF-κB mechanisms via TLR4 [16,29], which affects the expression of inflammatory mediators such as COX-2 and iNOS [29,30]. The activation of HO-1/Nrf-2 antioxidant pathways directly impacts the regulation of an inflammatory reaction, so we tested the effects of FF treatment on the expression of inflammatory proteins. As the results show in Figure 4B, the expression of inflammatory proteins activated by LPS/D-GalN injection was strongly repressed by FF treatment, whereas the antioxidant pathway was effectively activated by FF treatment. Therefore, 6 days of FF administration was sufficient to suppress severe liver damage in these mice induced by LPS/D-GalN injection and effectively regulated cytokine production and aminotransferase secretion.

Next, we investigated how FF affects the inflammatory reaction in endotoxin-stimulated macrophages. FF pretreatment at a non-toxic concentration strongly inhibited the secretion of NO, IL-6, and IL-1β in RAW 264.7 cells upon LPS stimulation (Figure 5A–C) and suppressed the expression of the inflammatory enzyme iNOS (Figure 5D). Furthermore, the production of HO-1 was induced both when the FF was administered alone and in combination with LPS treatment (Figure 5D,F). In addition, Nrf-2 was activated by FF treatment and translocated to the nucleus (Figure 5E). In addition, Nrf-2 activation by FF was also observed under LPS stimulation (Figure 5F). The anti-inflammatory effects of FF in the macrophage cell line were replicated in primary mouse macrophages, and pretreatment with FF inhibited the secretion of various inflammatory mediators in PMC in a pattern similar to those observed in RAW 264.7 macrophages (Figure 6). Taken together, FF effectively alleviated fulminant liver injury in these mice, and its efficacy is believed to be associated with a powerful anti-inflammatory activity.

Subsequently, to investigate the relationship between the physiological activities of FF and its constituents, we performed phytochemical analyses using HPLC. Under HPLC-DAD analysis conditions, we separated and identified the three main components including forsythiaside A, pinoresinol, and phillygenin (Figure 1). Previous studies indicated that forsythiaside A exerts protective effect against LPS/D-GalN-induced liver injury in mice via inhibiting NF-κB activation and up-regulating Nrf-2/HO-1 [31]. Similarly, forsythiaside A shows hepatoprotective effect against acetaminophen-induced liver injury in zebrafish through regulation of TNF, matrix metallopeptidase (MMP)9, MMP2, and phosphatidylinositol 3-kinase [32]. In addition, forsythiaside A exhibits anti-inflammatory and antioxidant efficacy in BV2 microglia cells through activation of Nrf-2 and HO-1 signaling pathway [33]. Another previous study has shown that pinoresinol has hepatoprotective effect against carbon tetrachloride (CCl_4_)-induced hepatic damage in mice [34]. In addition, phillygenin inhibits fibrosis by LPS in human hepatic stellate cell LX2 [35] and shows hepatoprotective effect on CCl_4_-induced liver injury in mice by its antioxidant activity and inhibition on cytochrome P450 2E1 [36]. As can be seen from the results of previous studies mentioned above, several bioactive components of FF exhibit hepatoprotective, anti-inflammatory, and antioxidant effects. Based on our HPLC analysis and the results of previous studies on these constituents, the hepatoprotective, anti-inflammatory, and antioxidant effects of FF can likely reflect the presence of forsythiaside A, pinoresinol, and phillygenin.

## 5. Conclusions

In summary, this work demonstrated that FF mitigates LPS/D-GalN-induced fulminant liver injury in mice. FF strongly lowered the levels of inflammatory cytokines and aminotransferase in mouse serum and inhibited the expression of hepatic cytokine mRNAs. Furthermore, FF effectively ameliorates a strong inflammatory reaction and activates antioxidant mechanisms, thereby inhibiting hemorrhage and necrosis in liver tissue, significantly alleviating liver damage. The anti-inflammatory activities of FF have also been proved in experimental inflammatory models using a murine macrophage cell line and primary cells. Based on these results, FF is potentially valuable as a candidate to prevent or treat intense inflammation and resulting liver damage.

## Figures and Tables

**Table 1:** Primer sequences used for RT-qPCR.

Target Gene	Primer Sequence
TNF-α	F: 5′-TTCTGTCTACTGAACTTCGGGGTGATCGGTCC-3′
	R: 5′-GTATGAGATAGCAAATCGGCTGACGGTGTGGG-3′
IL-6	F: 5′-TCCAGTTGCCTTCTTGGGAC-3′
	R: 5′-GTGTAATTAAGCCTCCGACTTG-3′
IL-1β	F: 5′-ATGGCAACTGTTCCTGAACTCAACT-3′
	R: 5′-CAGGACAGGTATAGATTCTTTCCTTT-3′
β-actin	F: 5′-AGAGGGAAATCGTGCGTGAC-3′
	R: 5′-CAATAGTGATGACCTGGCCGT-3′

F, forward; R, reverse.

**Table 2:** Various antibodies used for Western blot.

Antibody	Corporation	Product No.	RRID	Dilution Rate
iNOS	Cell Signaling	#13120	AB_2687529	1:1000
COX-2	Cell Signaling	#4842	AB_2085144	1:1000
HO-1	Cell Signaling	#82206	AB_2799989	1:1000
Nrf-2	Cell Signaling	#12721	AB_2715528	1:1000
P-NF-κB p65	Cell Signaling	#3033	AB_331284	1:1000
P-IκBα	Cell Signaling	#2859	AB_561111	1:1000
IκBα	Cell Signaling	#4814	AB_390781	1:1000
P-ERK	Cell Signaling	#4377	AB_331775	1:1000
ERK	Cell Signaling	#9102	AB_330744	1:1000
P-p38	Cell Signaling	#9211	AB_331641	1:1000
p38	Cell Signaling	#9212	AB_330713	1:1000
P-JNK	Cell Signaling	#9251	AB_331659	1:1000
JNK	Cell Signaling	#9252	AB_2250373	1:1000
β-actin	Cell Signaling	#4970	AB_2223172	1:1000
TBP	Cell Signaling	#8515	AB_10949159	1:1000
2nd anti-mouse	Cell Signaling	#7076	AB_330924	1:5000
2nd anti-rabbit	Cell Signaling	#7074	AB_2099233	1:5000

**Table 3:** HPLC conditions for analysis of compounds and FF.

HPLC Conditions			
Detector	280 nm		
Column	X bridge C18 Column (250 mm × 4.6 mm, 5 μm)		
Column temperature	40°C		
Injection volume	10 μL		
Flow rate	1.0 mL/min		
Mobile phase	Time (min)	A	B
A: 0.3% acetic acid in water B: MeOH	0.0	95	5
	8.0	70	30
	24.0	43	57
	39.0	40	60
	50.0	30	70
	60.0	0	100

**Table 4:** Calibration curves of compounds.

Compound	Range (μg/mL)	Regression Equation	*r* ^2^	LOD (μg/mL)	LOQ (μg/mL)
1	200.0~500.0	y = 0.2516x − 3.8826	0.9958	0.0527	0.1598
2	20.0~200.0	y = 0.1132x + 0.1922	0.9990	0.0879	0.2664
3	2.5~25.0	y = 0.1927x + 0.0909	0.9994	0.0517	0.1565

Forsythoside A (1); Pinoresinol (2); Phillygenin (3). LOD = 3.3 × σ/*S*. LOQ = 10 × σ/*S*. σ is the standard deviation of the intercept from the regression equation and *S* is the slope of the calibration curve.

## Data Availability

The data are contained within the article.

## Supplementary Materials

The following are available online at https://www.mdpi.com/article/10.3390/nu13082901/s1, Figure S1: UV chromatogram of each standard compounds and FF.
