# Supplementary figures and images for: Forsythia Fruit Prevents Fulminant Hepatitis in Mice and Ameliorates Inflammation in Murine Macrophages
Source: Nutrients. 2021 Aug 23;13(8):2901. doi: 10.3390/nu13082901 (PMC8399229; doi:10.3390/nu13082901)

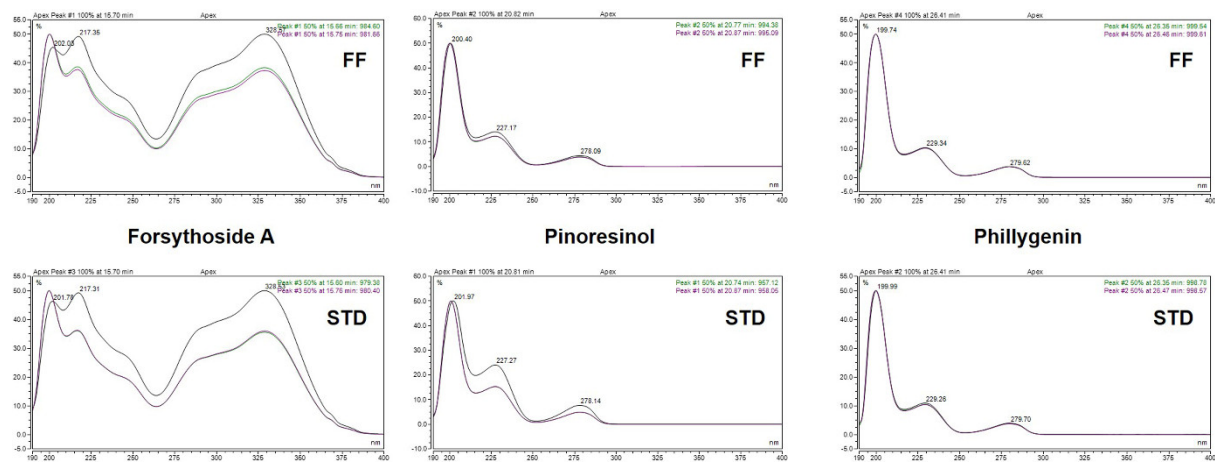

**Figure S1.** UV chromatogram of each standard compounds and FF.

Supplement: Supplementary file 1 [file nutrients-13-02901-s001.zip › nutrients-1340204-supplementary.pdf]
